# Periodic and Aperiodic Alterations of Resting‐State EEG in Schizophrenia Spectrum Disorders: Cognitive and Clinical Insights

**DOI:** 10.1111/ejn.70263

**Published:** 2025-10-04

**Authors:** Genc Hasanaj, Iris Jaeger, Berkhan Karsli, Enrico Schulz, Emanuel Boudriot, Lukas Roell, Maxim Korman, Marcel S. Kallweit, Fanny Dengl, Nicole Klimas, Kristin Fischer, Katharina Hanken, Verena Meisinger, Joanna Moussiopoulou, Vladislav Yakimov, Susanne Karch, Alkomiet Hasan, Andrea Schmitt, Peter Falkai, CDP Working Group, Oliver Pogarell, Florian J. Raabe, Elias Wagner, Matin Mortazavi, Daniel Keeser

**Affiliations:** ^1^ Department of Psychiatry and Psychotherapy LMU University Hospital, LMU Munich Munich Germany; ^2^ NeuroImaging Core Unit Munich (NICUM), LMU University Hospital, LMU Munich Munich Germany; ^3^ Evidence‐Based Psychiatry and Psychotherapy, Faculty of Medicine University of Augsburg Augsburg Germany; ^4^ Department of Radiology LMU University Hospital, Ludwig‐Maximilians‐Universität München Munich Germany; ^5^ Department of Medical Psychology Ludwig‐Maximilians‐Universität München Munich Germany; ^6^ Max Planck Institute of Psychiatry Munich Germany; ^7^ International Max Planck Research School for Translational Psychiatry (IMPRS‐TP) Germany; ^8^ Department of Psychiatry, Psychotherapy and Psychosomatics, Medical Faculty University of Augsburg, BKH Augsburg Augsburg Germany; ^9^ German Center for Mental Health (DZPG), Partner Site Munich/Augsburg Munich Germany; ^10^ Laboratory of Neuroscience (LIM27), Institute of Psychiatry University of São Paulo São Paulo Brazil; ^11^ Munich Center for Neurosciences (MCN), LMU Munich, Planegg Martinsried Germany

**Keywords:** aperiodic, cognition, EEG, frontoinsular, periodic, schizophrenia spectrum disorders

## Abstract

Schizophrenia spectrum disorders (SSD) are marked by cognitive deficits and clinical symptoms linked to disrupted neural oscillations. While changes in spectral power are well documented in SSD, many studies have not clearly separated rhythmic (periodic) from the nonrhythmic (aperiodic) brain activity. This study examined both periodic and aperiodic resting‐state EEG components in SSD, recorded from 152 healthy controls and 97 SSD participants. EEG features (periodic power, bandwidth, center frequency; aperiodic exponent and offset) were extracted from global scalp averages and frontoinsular regions, including the dorsal anterior cingulate cortex (dACC), right anterior insula (R‐INS), and left anterior insula (L‐INS). At the scalp level, SSD individuals exhibited a global increase in theta power, along with a decreased alpha center frequency. Aperiodic activity showed increased exponent and offset in SSD. In frontoinsular regions, increased theta power was observed in the dACC, R‐INS, and L‐INS, along with lower alpha center frequency in L‐INS. No significant differences were found for aperiodic activity in these regions. Increased frontoinsular theta power, especially in the dACC, was associated with worse cognitive performance, particularly global cognition and working memory. These findings highlight the importance of separating periodic and aperiodic EEG activity in SSD, suggesting that periodic alterations, particularly in frontoinsular theta oscillations, may underlie cognitive dysfunction in SSD.

## Graphical abstract

This study examined periodic and aperiodic EEG activity in schizophrenia spectrum disorder (SSD) using FOOOF across scalp and source‐level recordings. SSD patients showed increased theta power and aperiodic components, with dACC theta predicting cognitive impairment. Findings highlight altered excitation–inhibition dynamics and emphasize the utility of spectral parameterization in SSD.

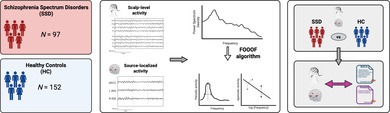

**Abbreviations**
BACS: brief assessment of cognition in schizophrenia
CPZeq: chlorpromazine equivalent
dACC: dorsal anterior cingulate cortex
EC: eyes closed
EEG: electroencephalography
EO: eyes open
FOOOF: fitting oscillations and one over F
HC: healthy controls
L‐INS: left anterior insula
PANSS: positive and negative syndrome scale
R‐INS: right anterior insula
SSD: schizophrenia spectrum disorders

## 1 Introduction

Understanding the neural basis of cognitive and clinical symptoms in schizophrenia spectrum disorders (SSDs) remains one of the most pressing challenges in clinical neuroscience (Kahn et al. 2015; McCutcheon, Reis Marques, et al. 2020). Cognitive impairments in SSD emerge early and affect multiple aspects of personal and social functioning, with general cognitive performance being 1.5 standard deviations lower compared to a healthy control (HC) population (McCutcheon, Keefe, et al. 2023). These impairments are closely linked to structural and functional brain alterations, driving ongoing efforts to identify markers that can offer closer insights into disease status and progression (Gordillo et al. 2023; Porter et al. 2023).

There is abundant evidence suggesting widespread structural and functional abnormalities across mental disorders (Dong et al. 2018; Picó‐Pérez et al. 2022; Sha et al. 2019). Of particular importance, Goodkind et al. (2015) identified the dorsal anterior cingulate cortex (dACC) and bilateral anterior insula as three brain regions commonly affected across both psychotic and nonpsychotic mental illnesses, suggesting that these frontoinsular regions may serve as transdiagnostic vulnerability hubs. More recently, a large‐scale network‐based study reinforced the centrality of these frontoinsular regions, showing that they not only exhibit consistent alterations but also act as disease epicenters in schizophrenia, from which structural and functional pathology appears to propagate (Jiang et al. 2024). In contrast to other brain regions (e.g., the hippocampus or temporoparietal regions), which show greater inter‐individual variability, the frontoinsular alterations emerge as consistently altered and spatially central in schizophrenia (Jiang et al. 2024). Moreover, altered activity within these regions has been linked to impaired salience processing, which may contribute to the origin and maintenance of cognitive and clinical symptoms in SSD (Menon et al. 2023).

Neural oscillations provide a reliable index of brain functions and play a crucial role in coordinating communication between brain regions (Hirano and Uhlhaas 2021). Disruptions in these oscillatory mechanisms are increasingly regarded as potential markers of both cognitive and symptomatic impairments in SSD (Günther and Hanganu‐Opatz 2022; Phillips and Uhlhaas 2015). Altered activity across both low‐ and high‐frequency bands is widely reported, specifically amplitudes including theta and gamma frequency bands in first‐episode, unmedicated, and chronic schizophrenia individuals during resting‐state EEG (Hirano et al. 2015; Hirano and Uhlhaas 2021). While alpha power differences in SSD are less stable, alpha peak power frequency is notably reduced in SSD and is closely associated with cognitive dysfunctions (Catalano et al. 2024; Sponheim et al. 2023).

Besides oscillatory (periodic) brain activity, the brain also generates nonrhythmic (aperiodic) activity that follows a characteristic 1/f‐like spectral distribution (Donoghue et al. 2020; He 2014). This activity is thought to reflect the brain's excitation and inhibition (E/I) balance, as shown in both in vivo and in silico models (Gao et al. 2017; Wiest et al. 2023), and its modulation has been associated with improvements in symptoms and cognition in SSD and major depression (Molina et al. 2020; Smith et al. 2023). However, findings on group differences between SSD and HC remain mixed, with reports of increased, decreased, or unchanged aperiodic components, potentially due to differences in study design (Donoghue 2024). The contribution of resting‐state periodic and aperiodic activity to pathophysiology and clinical symptoms still remains unclear, particularly within the epicenters of SSD.

This study aimed to investigate how periodic and aperiodic resting‐state EEG activities are altered in SSD, both at the scalp level and within frontoinsular regions, and how these alterations relate to cognitive functioning and symptom severity. The existing literature is limited, lacking insight into how these components differ between individuals with SSD and healthy controls, particularly within frontoinsular regions that are central to SSD pathophysiology. Given their transdiagnostic vulnerability, centrality in schizophrenia‐related network pathology, and involvement in salience processing, we selected the bilateral anterior insula and dACC as a priori regions of interest. These regions were chosen to examine whether alterations in oscillatory and aperiodic EEG activity in SSD converge on structurally and functionally critical neural hubs. For anatomical precision, we used ROI masks derived from the original Goodkind et al. (2015) study. We first compared the global scalp‐level periodic activity (power, bandwidth, and center frequency) across theta, alpha, beta, and low‐gamma frequency bands, along with aperiodic activity quantified as exponent and offset, and sought to resolve discrepancies in the literature using a relatively large cohort. We repeated the same analyses for the frontoinsular regions, expecting alterations based on previous evidence of functional and structural changes in these areas. Subsequently, we examined how these alterations relate to cognitive performance as well as symptom severity in SSD.

## 2 Materials and Methods

### 2.1 Study Sample and Design

The sample for this study is part of the Munich Clinical Deep Phenotyping study (Krčmář et al. 2023), which was approved by the local ethics committee of LMU Munich (approval numbers: 20‐528 and 22‐0035) and registered in the German Clinical Trials Register (DRKS, registration ID: DRKS00024177). Participants included both inpatients and outpatients who were recruited at the Department of Psychiatry and Psychotherapy, LMU University Hospital, Munich, Germany. HC subjects were recruited from the local community by advertising online and in public places. Inclusion criteria for patients were a diagnosis of schizophrenia, schizoaffective disorder, brief psychotic disorder, unspecified schizophrenia spectrum disorder (SSD), and delusional disorder, according to the German version 7.0.2 of the MINI International Neuropsychiatric Interview (Sheehan et al. 1998). HC participants were screened using the same diagnostic interview and excluded if any current or past mental disorder was suspected.

All of the participants were assessed with the Positive and Negative Syndrome Scale (PANSS) (Kay et al. 1987). Cognitive performance was assessed using the German version of the Brief Assessment of Cognition in Schizophrenia (BACS), with scores standardized as z‐scores using the HC group as reference (Keefe et al. 2004; Sachs et al. 2011). All clinical interviews and questionnaires were performed by trained personnel. Information about current medication, such as antipsychotic medication and benzodiazepine use, was obtained from medical records and self‐report of the participants. Antipsychotic medications were converted (when applicable) to common chlorpromazine equivalent doses (CPZeq) based on the defined daily doses (DDD) method (Leucht et al. 2016).

### 2.2 Magnetic Resonance Imaging and Preprocessing

To increase the precision of EEG source localization, anatomical MRI T1w MPGRAGE and T2w images were recorded to provide individual structural details, enabling a more accurate mapping of electrical activity to specific brain regions (Michel and Brunet 2019). MRI scans were conducted using the Siemens MAGNETOM Prisma 3 T scanner (Siemens Healthineers AG) with a 32‐channel head coil at the NeuroImaging Core Unit Munich (NICUM). The T1‐weighted images were obtained through a magnetization‐prepared rapid gradient‐echo sequence. These images featured an isotropic voxel size of 0.8 mm^3^, encompassing 208 slices. The scanning parameters included a repetition time of 2500 ms, an echo time of 2.22 ms, a flip angle of 8°, and a field of view measuring 256 mm^2^.

### 2.3 EEG Recording and Preprocessing

Resting state EEG was recorded using 32 scalp electrodes (Electro‐Cap International Inc., Eaton, OH, USA) following the International 10–20 system. The recording was conducted using the BrainAmp amplifier (Brain Products, Martinsried, Germany), at a sampling rate of 1000 Hz, with Cz used as the reference electrode. Electrode skin impedance was kept below 5 kΩ. In total, the recording of the resting state EEG lasted 10 min, with the first 5 min recorded during eyes closed (with eyes opening for 3 s after 120 s to suppress excessive alpha power) and the last 5 min of eyes open. Participants were instructed to remain as calm and relaxed as possible throughout the recording.

The raw data were preprocessed using an automated in‐house preprocessing pipeline implemented in MATLAB (The Mathworks Inc.) using EEGLAB v2022.0 (Delorme and Makeig 2004) (https://sccn.ucsd.edu/eeglab/). Each resting‐state condition was preprocessed separately for each participant. All the data were initially re‐referenced to the mastoid electrodes (A1 and A2) and resampled down to 256 Hz. A bandpass filter (1–70 Hz) and a notch filter were applied, and data were segmented into 6‐s epochs.

Each epoch was further preprocessed to remove linear trends, filtered on low and high‐frequency bands. Channels were rejected based on extreme power spectrum values, kurtosis, and joint probability. Preprocessing included Independent Component Analysis (ICA), and detected artifact components were removed using the Multiple Artifact Rejection Algorithm (MARA) (Winkler et al. 2011). After ICA, the data removal and channel rejection are reapplied with minor modifications. The removed channels were interpolated at the final stage of preprocessing (for details, see Supporting Information).

### 2.4 Source Localization of EEG Activity

Cortical source activations were estimated using Brainstorm software (version 15‐Nov‐2024), an open‐source brain imaging tool (Tadel et al.^2011^); (http://neuroimage.usc.edu/brainstorm). For each subject, their respective anatomical data were imported, and a three‐layer boundary element method (BEM) was applied to compute the lead field matrix. The head model was computed using the OpenMEEG BEM method, incorporating scalp, skull, and brain conductivities with adaptive integration. Source localization was performed using the Linearly Constrained Minimum Variance (LCMV) beamformer method, with the inverse solution computed for a Pseudoinverse of the Noise‐normalized Average Inverse (PNAI) measure using free orientation and depth weighting. Source localized activity was extracted across three frontoinsular regions of interest: the right anterior insula (R‐INS), the left anterior insula (L‐INS), and the dACC, based on the masks from Goodkind et al. (2015) meta‐analysis (for details of source‐localized parameters see Supporting Information).

### 2.5 Parameterization of Power Spectrum Density

The power spectral density (PSD) was calculated using the Welch method, with 6‐s windows and 50% overlap on MNE Python (v1.8.0) (Gramfort et al. 2014). PSD was calculated for all electrodes and for the three source‐localized ROIs (R‐INS, L‐INS, dACC). To parameterize the PSD, the *fooof* Python toolbox was used (https://fooof‐tools.github.io/fooof/) (Donoghue et al. 2020). The power spectra were parameterized across a frequency range of 3–40 Hz, and the final model parameters included a model with: peak_width_limits = [1, 10], maximum_n_peak = 8, min_peak_height = 0.1, peak_threshold = 2, and aperiodic_mode = “fixed”. The fooof model demonstrated good fitting performance across both groups and different extractions, as indicated by *R* ^2^ values and error in parentheses: Scalp‐level—HC (0.963 ± 0.057), SSD (0.967 ± 0.062); L‐INS—HC (0.957 ± 0.054), SSD (0.960 ± 0.056); R‐INS—HC (0.958 ± 0.0544), SSD (0.959 ± 0.057); dACC—HC (0.959 ± 0.055), SSD (0.962 ± 0.056). All the subsequent analyses in the Result section refer to the aperiodic‐adjusted power, center frequency, and bandwidth.

The FOOOF algorithm decomposes the PSD into aperiodic and periodic components; it first fits the aperiodic background activity and subtracts it from the original spectrum to isolate residuals that may contain oscillatory peaks (Donoghue et al. 2020). These peaks are then identified based on user‐defined thresholds (e.g., minimum peak height and standard deviation threshold) and modeled using Gaussian functions. The final model is constructed by summing the fitted aperiodic component with the Gaussian functions representing the detected peaks, providing a full parameterization of the power spectrum. Due to the specific detection thresholds applied in this study and the relatively lower power of peaks outside the alpha and beta bands, reliable periodic peaks were less frequently detected in the theta and gamma ranges (see Figures S1 and S2).

At scalp level activity, periodic and aperiodic features were averaged across all electrodes. Considering that the FOOOF algorithm's peak detection can result in missing values (NAs) for the signal on which no peak is detected, we set an inclusion/exclusion threshold: if at least 50% of electrodes in a given frequency band exhibited periodic activity, the activity was averaged across all active electrodes. Otherwise, the participants were labeled as missing. At source level, participants were similarly labeled as missing if no periodic activity was detected in a given region (R‐INS, L‐INS, dACC). All statistical analyses were conducted only on available (non‐missing) data.

### 2.6 Statistical Analyses

All the statistical analyses were performed in R, version 4.4.0 (R Core Team 2024). Fisher's exact test was used to show differences between categorical variables, while Welch's *t*‐test was applied to continuous variables when no covariates were included. To investigate group differences in periodic and aperiodic activity on source and scalp levels, we performed Linear Mixed‐Effects Models (LMM) using the *lmer* function in the lme4 package (Bates et al. 2015). The dependent variables were the periodic and aperiodic components, while the primary predictor was group status (HC vs. SSD). To account for potential interactions between condition (eyes closed vs. eyes open) and group status, we included condition as an interaction term (Zhang et al. 2021). Sex and age were included as covariates, and participant ID was modeled as a random intercept to account for inter‐individual variability.

Separate models were run for the scalp‐level activity averaged across all electrodes and three ROI source localized activities (R‐INS, L‐INS, dACC), for three periodic parameters (power, bandwidth, center frequency), five frequency bands (theta: 4–8 Hz, alpha: 8–12 Hz, beta: 12–30 Hz, low‐gamma/gamma1: 30–40 Hz), as well as two aperiodic components (exponent and offset). For the scalp level, for each extraction modality (scalp‐level and source‐level), *p* values were adjusted for multiple comparisons using the Benjamini–Hochberg procedure for all the models run (Benjamini and Hochberg 1995). The corrected *p* values are further referred to as *q* values.

Linear regressions are used to examine the relationships between altered EEG components for each condition (eyes closed and eyes open) with cognitive and symptomatic measures while controlling for age, sex, and CPZeq.

## 3 Results

### 3.1 Cohort Characteristics

The sample considered for this study consisted of 97 individuals with SSD (28.9% female, mean age = 38.2 ± 11.8, PANSS total score = 56.6 ± 16.7, illness duration (months) = 130.45 ± 123.51, chlorpromazine equivalents = 329.10 ± 273.33 mg, 16.5% benzodiazepine users) and 152 HC (55.9% female, mean age = 34.79 ± 12.69). The two most prevalent diagnoses in the SSD group were schizophrenia (53.8%) and schizoaffective disorder (27.9%). 38.54% of our SSD sample was in remission according to modified Andreasen criteria without the time criterion (Andreasen et al. 2005). Table 1 provides more details on the clinical and demographic characteristics of the cohorts.

**TABLE 1:** Sample characteristics.

	SSD		HC		
	Mean ± SD or *n* (%)	*n*	Mean ± SD or *n* (%)	*n*	*p*
Demographic characteristics					
Age, years	38.2 ± 11.8	97	34.8 ± 12.7	152	0.004^b^
Sex, female:male (% female)	26:68 (28.9%)	97	85:67 (55.9%)	152	< 0.001^a^
BMI	27.8 ± 5.44	95	23.4 ± 3.6	147	< 0.001^b^
Disease characteristics					
Disease duration, months	130.45 ± 123.51	93			
Antipsychotic treatment duration, months	105.20 ± 116.18	89			
Benzodiazepine use, yes:no (% yes)	16:81 (16.5%)	97			
CPZeq, mg	329.10 ± 273.33	94			
Remission Andreasen, yes:no (% yes)	37:59 (38.54%)	97			
PANSS Positive symptoms	13.0 ± 5.09	96	7.30 ± 0.76	152	< 0.001^b^
PANSS Negative symptoms	13.8 ± 5.64	96	7.39 ± 0.88	152	< 0.001^b^
PANSS General symptoms	29.80 ± 8.78	96	16.9 ± 1.43	152	< 0.001^b^
PANSS Total score	56.60 ± 16.70	96	31.59 ± 2.43	152	< 0.001^b^
GAF	53.12 ± 12.83	97	89.88 ± 6.14	152	< 0.001^b^
BACS Composite z score	−1.90 ± 1.42	91	0 ± 1	148	< 0.001^b^
Diagnosis					
Schizophrenia	56 (57.73%)				
Schizoaffective disorder	29 (29.9%)				
Brief Psychotic disorder	7 (7.22%)				
Unspecified schizophrenia Spectrum disorder	3 (3.09%)				
Delusional disorder	2 (2.06%)				

Abbreviations: BACS = Brief Assessment of Cognition in Schizophrenia; BMI = body mass index; CPZeq = chlorpromazine equivalent dose; GAF = Global Assessment of Functioning; HC = healthy control participant; n = number of participants; p = p‐value for the respective test; PANSS = Positive and Negative Syndrome Scale; SD = standard deviation; SSD = schizophrenia spectrum disorder.

^a^ Fisher's exact test.

^b^ Welch's *t* test.

### 3.2 Periodic and Aperiodic Effects on Scalp Level Activity

**FIGURE 1:**
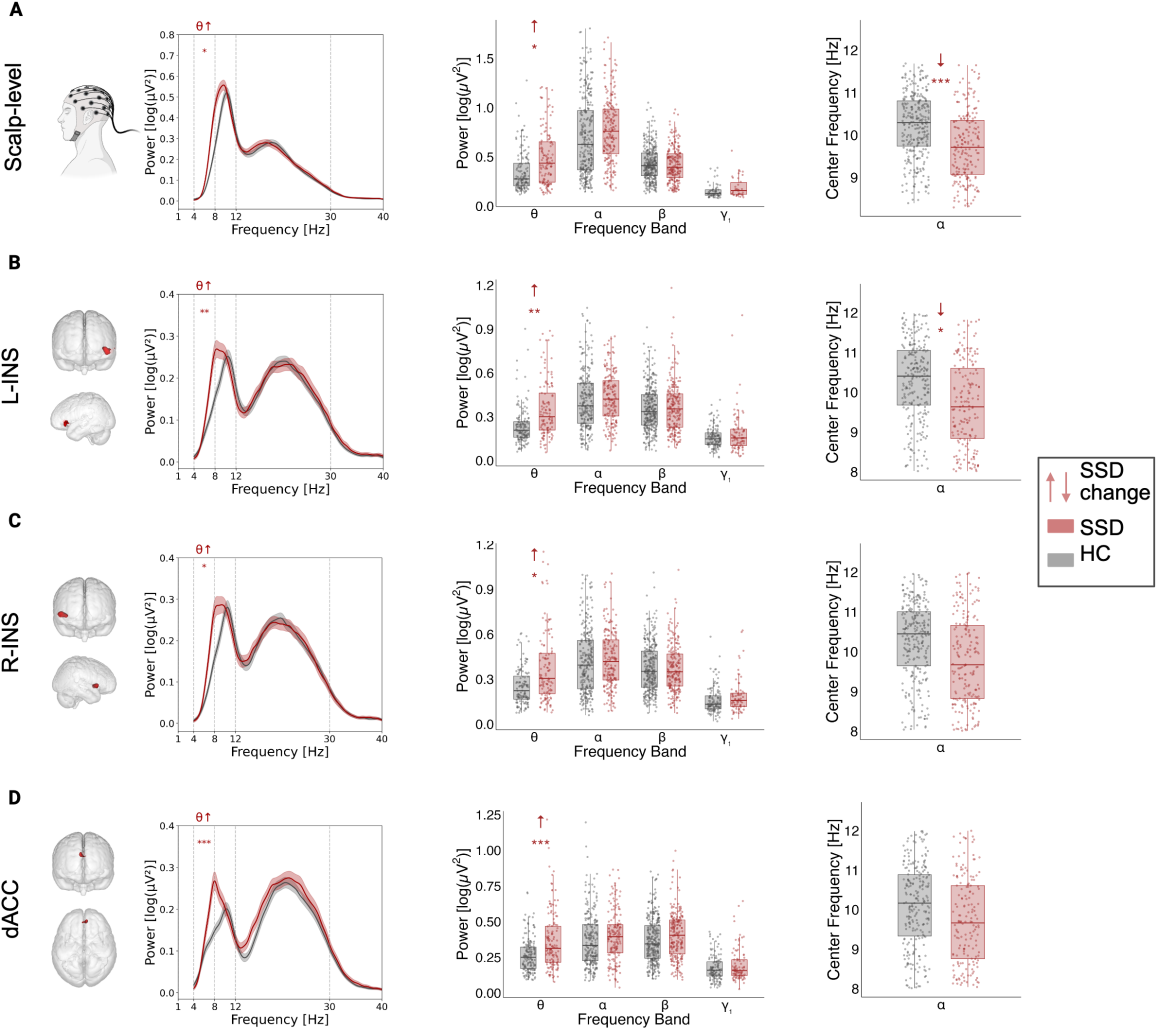
Differences in periodic activity at scalp‐level and source‐localized regions. For (A) scalp‐level, (B) left anterior insula, (C) right anterior insula, and (D) dorsal anterior cingulate cortex: The first column visually illustrates the origin of the extracted activity and shows the periodic power spectral density (PSD) for each group; the second column shows power comparisons between HC and SSD across frequency bands; and the third column shows alpha center frequency comparisons between HC and SSD. HC = healthy controls. SSD = schizophrenia spectrum disorder. Scalp‐level: averaged activity across electrodes. L‐INS: left anterior insula. R‐INS: right anterior insula. dACC: dorsal anterior cingulate cortex. ↓↑: show an increase or decrease in SSD compared to HC. Significance level after FDR correction: *< .05, **< .01, ***< .001.

**FIGURE 2:**
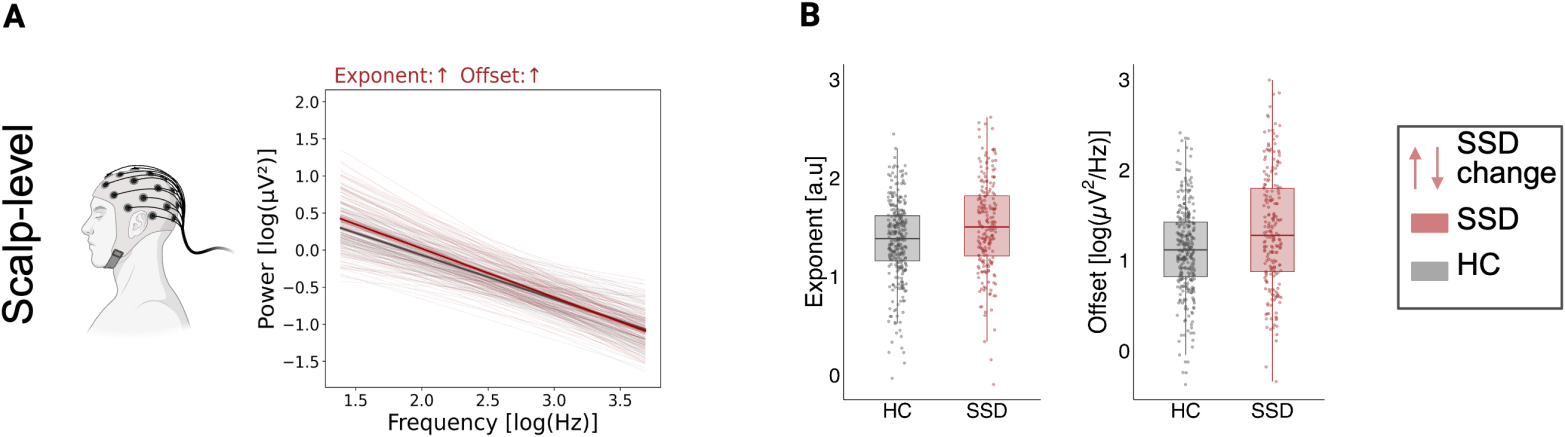
Global scalp‐level differences on aperiodic component. (A) Aperiodic components of the power spectrum (bold) averaged across individuals within each diagnostic group. (B) Comparison between HC and SSD for aperiodic exponent and offset shows that these two groups are significantly increased in the SSD group after controlling for age and sex (*q* > 0.05). HC = healthy controls. SSD = schizophrenia spectrum disorder. Scalp‐level: averaged activity across electrodes. ↓↑: show an increase or decrease in the SSD group. Significance level after FDR correction: *< .05, **< .01, ***< .001.

To explore whether periodic and aperiodic activity differed between HC and SSD, we applied linear mixed models. At scalp level, for power as a component of periodic activity, we observed an increase in theta power (*b* = 0.108 log (μV^2^), 95% CI [0.029, 0.187]; *p* < 0.008, *q* = 0.027) in the SSD group compared to HC. Conversely, SSD individuals showed pronounced reductions in the alpha center frequency peak (*b* = −0.435 Hz, 95% CI [−0.642, −0.229]; *p* < 0.001, *q* < 0.001) (see Figure 1 and Table 2). In terms of aperiodic activity, the SSD group demonstrated increased exponent (*b* = 0.168 a.u., 95% CI [0.056, 0.281]; *p* = 0.004, *q* = 0.017) and offset (*b* = 0.272 log (μV^2^/Hz), 95% CI [0.126, 0.418]; *p* < 0.001, *q* = 0.002) relative to HC (see Figure 2 and Table 2).

**TABLE 2:** Linear Mixed‐Effects models of comparing periodic and aperiodic components between healthy controls and schizophrenia spectrum disorders.

Region	Frequency band	FOOOF parameter	N HC (EC:EO)	N SSD (EC:EO)	Estimates [CI95%: LL, UL]	*p*	*q* (p_FDR_)	*R* ^2^ (Marginal)	*R* ^2^ (Conditional)
Scalp‐level	Theta: 4–8 Hz	Bandwidth	90 (68:70)	75 (58:62)	0.0352 [−0.1159, 0.1862]	0.6474	0.6972	0.065	0.3677
Scalp‐level	Theta: 4–8 Hz	Power	90 (68:70)	75 (58:62)	0.1083 [0.0292, 0.1874]	0.0076	0.0266	0.1493	0.8411
Scalp‐level	Theta: 4–8 Hz	Center frequency	90 (68:70)	75 (58:62)	0.0877 [−0.1238, 0.2998]	0.4156	0.5818	0.0461	0.7682
Scalp‐level	Low‐gamma: 30–40 Hz	Bandwidth	39 (24:35)	27 (18:20)	−0.2541 [−0.9593, 0.4555]	0.4764	0.6063	0.0156	0.7392
Scalp‐level	Low‐gamma: 30–40 Hz	Power	39 (24:35)	27 (18:20)	0.0529 [0.005, 0.1008]	0.0313	0.0876	0.0523	0.7387
Scalp‐level	Low‐gamma: 30–40 Hz	Center frequency	39 (24:35)	27 (18:20)	0.7862 [−0.5053, 2.0778]	0.231	0.5028	0.0325	0.7815
Scalp‐level	Alpha: 8–12 Hz	Bandwidth	150 (149:144)	96 (96:90)	−0.0299 [−0.1451, 0.0853]	0.6107	0.6972	0.0701	0.5333
Scalp‐level	Alpha: 8–12 Hz	Power	150 (149:144)	96 (96:90)	0.0423 [−0.0494, 0.1341]	0.3652	0.5681	0.1755	0.6255
Scalp‐level	Alpha: 8–12 Hz	Center frequency	150 (149:144)	96 (96:90)	−0.4351 [−0.6416, −0.2287]	< 0.0001	< 0.0001	0.1132	0.7259
Scalp‐level	Beta: 12–30 Hz	Bandwidth	152 (152:152)	97 (97:97)	−0.1308 [−0.3978, 0.1362]	0.3362	0.5681	0.0978	0.6603
Scalp‐level	Beta: 12–30 Hz	Power	152 (152:152)	97 (97:97)	−0.0252 [−0.0683, 0.0179]	0.2514	0.5028	0.1014	0.7571
Scalp‐level	Beta: 12–30 Hz	Center frequency	152 (152:152)	97 (97:97)	−0.1257 [−0.9075, 0.656]	0.7521	0.7521	0.0189	0.7799
Scalp‐level	Aperiodic: 3–40 Hz	Exponent	152 (152:152)	97 (97:97)	0.1684 [0.0557, 0.2812]	0.0036	0.0168	0.0558	0.7757
Scalp‐level	Aperiodic: 3–40 Hz	Offset	152 (152:152)	97 (97:97)	0.2719 [0.1257, 0.4182]	0.0003	0.0021	0.0677	0.8708
L‐INS	Theta: 4–8 Hz	Bandwidth	92 (73:68)	71 (58:48)	−0.0931 [−0.4099, 0.2238]	0.5634	0.6761	0.0182	0.2231
L‐INS	Theta: 4–8 Hz	Power	92 (73:68)	71 (58:48)	0.1034 [0.0468, 0.16]	0.0004	0.0084	0.119	0.734
L‐INS	Theta: 4–8 Hz	Center frequency	92 (73:68)	71 (58:48)	0.0418 [−0.2331, 0.3162]	0.7647	0.7647	0.0464	0.3538
L‐INS	Alpha: 8–12 Hz	Bandwidth	134 (127:118)	88 (78:81)	−0.088 [−0.3486, 0.1727]	0.5076	0.6662	0.034	0.1063
L‐INS	Alpha: 8–12 Hz	Power	134 (127:118)	88 (78:81)	0.0104 [−0.0439, 0.0645]	0.7073	0.7246	0.0207	0.4504
L‐INS	Alpha: 8–12 Hz	Center frequency	134 (127:118)	88 (78:81)	−0.4295 [−0.7191, −0.1405]	0.0037	0.0389	0.1191	0.5443
L‐INS	Beta: 12–30 Hz	Bandwidth	152 (151:151)	97 (97:95)	−0.4531 [−0.9285, 0.0224]	0.0619	0.2363	0.0243	0.2067
L‐INS	Beta: 12–30 Hz	Power	152 (151:151)	97 (97:95)	−0.0111 [−0.0514, 0.0291]	0.5868	0.6846	0.0957	0.6547
L‐INS	Beta: 12–30 Hz	Center frequency	152 (151:151)	97 (97:95)	0.6339 [−0.5162, 1.784]	0.2795	0.5104	0.018	0.4985
L‐INS	Low‐gamma: 30–40 Hz	Bandwidth	88 (70:52)	52 (38:40)	0.4399 [−0.3509, 1.2301]	0.2681	0.5104	0.0504	0.0843
L‐INS	Low‐Gamma: 30–40 Hz	Power	88 (70:52)	52 (38:40)	0.0574 [0.0136, 0.1012]	0.0105	0.0882	0.0437	0.4839
L‐INS	Low‐gamma: 30–40 Hz	Center frequency	88 (70:52)	52 (38:40)	0.3319 [−0.9397, 1.6034]	0.6078	0.6899	0.0073	0.5068
L‐INS	Aperiodic: 3–40 Hz	Exponent	152 (152:152)	97 (97:97)	0.05 [−0.0264, 0.1264]	0.1996	0.4412	0.0661	0.5968
L‐INS	Aperiodic: 3–40 Hz	Offset	152 (152:152)	97 (97:97)	0.1941 [−0.0232, 0.4114]	0.08	0.2585	0.0384	0.4157
R‐INS	Theta: 4–8 Hz	Bandwidth	89 (61:63)	66 (54:51)	−0.1197 [−0.3936, 0.1541]	0.3905	0.5656	0.0154	0.3644
R‐INS	Theta: 4–8 Hz	Power	89 (61:63)	66 (54:51)	0.1013 [0.0351, 0.1675]	0.0029	0.0389	0.1309	0.6592
R‐INS	Theta: 4–8 Hz	Center frequency	89 (61:63)	66 (54:51)	0.0701 [−0.2354, 0.3759]	0.652	0.7166	0.0529	0.4071
R‐INS	Alpha: 8–12 Hz	Bandwidth	142 (135:129)	95 (91:84)	0.0982 [−0.1189, 0.3152]	0.3746	0.5619	0.0346	0.0644
R‐INS	Alpha: 8–12 Hz	Power	142 (135:129)	95 (91:84)	−0.0361 [−0.0909, 0.0187]	0.1963	0.4412	0.047	0.4658
R‐INS	Alpha: 8–12 Hz	Center frequency	142 (135:129)	95 (91:84)	−0.3137 [−0.591, −0.0363]	0.0268	0.1608	0.0705	0.4227
R‐INS	Beta: 12–30 Hz	Bandwidth	152 (152:152)	97 (96:96)	−0.1036 [−0.5735, 0.3664]	0.6654	0.7166	0.048	0.3714
R‐INS	Beta: 12–30 Hz	Power	152 (152:152)	97 (96:96)	−0.0393 [−0.0804, 0.0018]	0.061	0.2363	0.0805	0.6717
R‐INS	Beta: 12–30 Hz	Center frequency	152 (152:152)	97 (96:96)	0.5418 [−0.6479, 1.7308]	0.3713	0.5619	0.0192	0.5435
R‐INS	Low‐gamma: 30–40 Hz	Bandwidth	84 (56:60)	48 (35:35)	0.2592 [−0.5518, 1.0719]	0.5284	0.6725	0.0366	0.0473
R‐INS	Low‐gamma: 30–40 Hz	Power	84 (56:60)	48 (35:35)	0.0214 [−0.0194, 0.0621]	0.3004	0.5257	0.0627	0.4297
R‐INS	Low‐gamma: 30–40 Hz	Center frequency	84 (56:60)	48 (35:35)	0.7382 [−0.5279, 2.005]	0.2521	0.5104	0.0206	0.4627
R‐INS	Aperiodic: 3–40 Hz	Exponent	152 (152:152)	97 (97:97)	0.0437 [−0.0331, 0.1205]	0.2645	0.5104	0.111	0.6276
R‐INS	Aperiodic: 3–40 Hz	Offset	152 (152:152)	97 (97:97)	0.1665 [−0.0571, 0.3901]	0.1443	0.3565	0.0289	0.342
dACC	Theta: 4–8 Hz	Bandwidth	98 (80:79)	75 (61:65)	−0.2132 [−0.4824, 0.0564]	0.1202	0.3366	0.0326	0.276
dACC	Theta: 4–8 Hz	Power	98 (80:79)	75 (61:65)	0.1145 [0.0611, 0.1678]	< 0.0001	< 0.0001	0.0874	0.7362
dACC	Theta: 4–8 Hz	Center frequency	98 (80:79)	75 (61:65)	0.1116 [−0.1847, 0.4078]	0.4593	0.6223	0.0755	0.5519
dACC	Alpha: 8–12 Hz	Bandwidth	132 (118:113)	84 (78:73)	0.2534 [0.0179, 0.489]	0.0351	0.1666	0.0421	0.1324
dACC	Alpha: 8–12 Hz	Power	132 (118:113)	84 (78:73)	−0.0108 [−0.0643, 0.0427]	0.6925	0.7246	0.034	0.4567
dACC	Alpha: 8–12 Hz	Center frequency	132 (118:113)	84 (78:73)	−0.368 [−0.6799, −0.0563]	0.0209	0.1463	0.041	0.4003
dACC	Beta: 12–30 Hz	Bandwidth	152 (151:152)	97 (97:96)	0.2065 [−0.3009, 0.7138]	0.4246	0.5944	0.0258	0.2141
dACC	Beta: 12–30 Hz	Power	152 (151:152)	97 (97:96)	0.0185 [−0.0213, 0.0582]	0.3621	0.5619	0.1155	0.7332
dACC	Beta: 12–30 Hz	Center frequency	152 (151:152)	97 (97:96)	0.3358 [−0.755, 1.4266]	0.5456	0.674	0.0042	0.4513
dACC	Low‐gamma: 30–40 Hz	Bandwidth	89 (61:62)	60 (43:46)	−0.8126 [−1.5694, −0.0559]	0.0357	0.1666	0.09	0.09
dACC	Low‐gamma: 30–40 Hz	Power	89 (61:62)	60 (43:46)	0.0349 [−0.0029, 0.0726]	0.0704	0.2464	0.0376	0.5157
dACC	Low‐gamma: 30–40 Hz	Center frequency	89 (61:62)	60 (43:46)	0.8077 [−0.2398, 1.8588]	0.1303	0.342	0.0314	0.7475
dACC	Aperiodic: 3–40 Hz	Exponent	152 (152:152)	97 (97:97)	0.0671 [−0.0129, 0.1472]	0.1003	0.3009	0.0975	0.6511
dACC	Aperiodic: 3–40 Hz	Offset	152 (152:152)	97 (97:97)	0.1318 [−0.1373, 0.401]	0.3365	0.5619	0.0056	0.4318

***Note:*** *Region* refers to either the scalp‐level analysis or specific regions of interest (ROIs): left anterior insula (L‐INS), right anterior insula (R‐INS), and dorsal anterior cingulate cortex (dACC). *Frequency Band* specifies the spectral range analyzed. *FOOOF parameter* refers to the component extracted using the FOOOF algorithm: power, center frequency, bandwidth (periodic), or exponent/offset (aperiodic). *N HC (EC:EO)* and *N SSD (EC:EO)* represent the number of unique participants per group, with the number of recordings from the eyes closed (EC) and eyes open (EO) conditions in parentheses. *Estimates [CI95%: LL, UL]* refer to the model estimate and the lower (LL) and upper (UL) bounds of the 95% confidence interval. *P* indicates the uncorrected *p*‐value; *q* (P_FDR_) indicates the *p*‐value after false discovery rate correction. *R* ^2^ *(marginal)* reflects variance explained by fixed effects; *R* ^2^ *(conditional)* includes both fixed and random effects.

Regarding covariates, we found increased theta power (*b* = 0.075 log (μV^2^), 95% CI [0.036, 0.114]; *p* < 0.001, *q* < 0.001), as well as a higher exponent (*b* = 0.06 a.u., 95% CI [0.013, 0.106]; *p* = 0.012, *q* = 0.028), and offset (*b* = 0.147 log (μV^2^/Hz), 95% CI [0.101, 0.193]; *p* < 0.001, *q* < 0.001) in the eyes closed condition compared to eyes open. Advancing age was associated with higher theta power (*b* = 0.004 log (μV^2^), 95% CI [0.001, 0.007]; *p* = 0.006, *q* = 0.020), and decreased alpha center frequency peak (*b* = −0.01 Hz, 95% CI [−0.017, −0.002]; *p* = 0.010, *q* = 0.027). No interactions were found between group and condition for any of the periodic or aperiodic measures (all *q* > 0.05).

### 3.3 Periodic and Aperiodic Effects on Source Localized Activity

Significant differences in periodic activity were present across all regions of interest. The SSD group had higher theta power in the L‐INS (*b* = 0.103 log (μV^2^), 95% CI [0.047–0.16]; *p* < 0.001, *q* = 0.008), R‐INS (*b* = 0.101 log (μV^2^), 95% CI [0.035 to 0.168]; *p* = 0.003, *q* = 0.039), and dACC (*b* = 0.114 log (μV^2^), 95% CI [0.061–0.168]; *p* < 0.001, *q* < 0.001) compared to HC. In addition to theta power, the SSD group showed a slower alpha center frequency only in L‐INS (*b* = −0.43 Hz, 95% CI [−0.719 to −0.141]; *p* = 0.004, *q* = 0.039) (see Figure 1 and Table 2). No group differences were observed for either aperiodic exponent or offset in these regions (all *q* > 0.05). Among covariates, advancing age was associated with a lower alpha center frequency in L‐INS (*b* = −0.013 Hz, 95% CI [−0.023 to −0.004]; *p* = 0.008, *q* = 0.030). Similarly to scalp level, no interactions were found between group and condition for any of the periodic or aperiodic measures at source (all *q* > 0.05).

### 3.4 Medication Effects on Periodic and Aperiodic Activity

To examine whether medications affect the altered EEG components in SSD, we utilized two different Linear Mixed Models using different predictors: (i) chlorpromazine equivalent (CPZeq; continuous) and (ii) benzodiazepine use (categorical: yes/no). Higher CPZeq dosage is associated with a higher exponent (*b* = 0.128 a.u., 95% CI [0.041–0.216]; *p* = 0.005, *q* = 0.019) and a higher offset (*b* = 0.172 log (μV^2^/Hz), 95% CI [0.047–0.297]; *p* = 0.007, *q* = 0.019). No significant differences were found between benzodiazepine users and non‐users at scalp level and source localized activity (all *q* > 0.05).

### 3.5 Follow‐Up Analysis: Associations of Altered EEG Features and Cognitive or Clinical Symptoms in SSD

After identifying the main altered periodic and aperiodic components in SSD, we further examined whether these components predict cognition and symptom severity in this group using multiple linear regressions. This relationship was investigated separately for the eyes closed and eyes open conditions. Given that increased CPZeq was associated with increased aperiodic exponent and offset, we included CPZeq as an additional covariate alongside age and sex.

**FIGURE 3:**
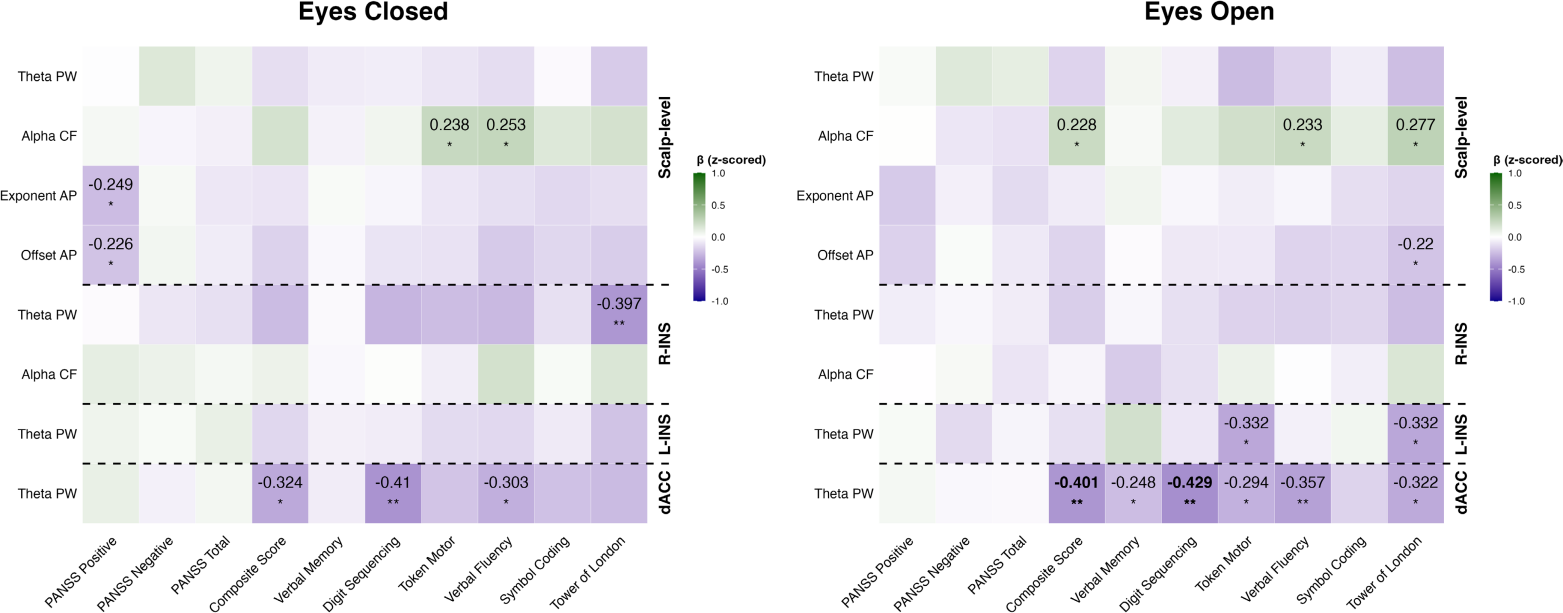
Heatmap of standardized regression estimates depicting the associations between altered EEG components in SSD (predictors) and cognitive performance (BACS) and symptom severity (PANSS) outcomes under eyes‐closed and eyes‐open conditions. All predictor and outcome variables were z‐scored for visualization purposes (refer to the main text for unstandardized estimates). Bolded values indicate results that survived FDR correction. SSD = Schizophrenia Spectrum Disorder; PW = Power; CF = Center Frequency; AP = Aperiodic. Significance level of the uncorrected *p*: * < .05, ** < .01, *** < .001.

Across both conditions, only two associations survived false discovery rate (FDR) correction. Specifically, increased theta power in the dACC during the eyes‐open condition was negatively associated with two cognitive outcomes: BACS composite score (*b* = −0.401, 95% CI [−0.642, −0.160], *p* = 0.002, *q* = 0.035) and digit sequencing (*b* = −0.429, 95% CI [−0.680, −0.178], *p* = 0.001, *q* = 0.035, *N* = 58). These findings suggest that enhanced frontal theta activity during wakeful rest may reflect impairments in cognitive control or working memory among SSD patients (see Figure 3).

In addition, we observed several uncorrected but potentially meaningful associations (*p* < 0.05, *q* > 0.05) between EEG features and cognitive or clinical variables. During the eyes‐closed condition, a higher aperiodic exponent at the scalp level was negatively associated with PANSS positive symptoms (*b* = −2.474, 95% CI [−4.47, −0.477], *p* = 0.016, *q* = 0.536, *N* = 93), as was aperiodic offset (*b* = −1.635, 95% CI [−3.107, −0.164], *p* = 0.030, *q* = 0.536, *N* = 93). These findings may indicate that stronger aperiodic activity, reflecting greater inhibitory tone or reduced E/I ratio, is linked to a less severe expression of positive symptoms.

In terms of cognition, alpha center frequency at the scalp during eyes closed was positively associated with token motor performance (*b* = 0.238, 95% CI [0.024, 0.452], *p* = 0.030, *q* = 0.249, *N* = 88) and verbal fluency (*b* = 0.253, 95% CI [0.039, 0.467], *p* = 0.021, *q* = 0.249, *N* = 88). During the eyes‐open condition, alpha CF remained positively associated with composite score (*b* = 0.228, 95% CI [0.01, 0.446], *p* = 0.040, *q* = 0.200, *N* = 82), verbal fluency (*b* = 0.233, 95% CI [0.012, 0.454], *p* = 0.039, *q* = 0.200, *N* = 82), and Tower of London (*b* = 0.277, 95% CI [0.065, 0.488], *p* = 0.011, *q* = 0.099, *N* = 80), suggesting that faster alpha oscillations may support better cognitive functioning in SSD. Additional uncorrected relationships involving source‐level theta power and other cognitive measures are visualized in Figure 3 and may warrant further investigation in future studies.

## 4 Discussion

This study reveals significant alterations in both periodic and aperiodic EEG activity in individuals with SSD. At scalp level, the SSD group showed a pronounced increase in theta power, alongside a reduction in alpha center frequency. Additionally, averaged scalp‐level activity showed elevated aperiodic exponent and offset in SSD compared to HC. Furthermore, frontoinsular regions showed a consistent increase of theta power in SSD, as well as decreased alpha center frequency at L‐INS, with no differences in aperiodic activity. In terms of the relationship between altered periodic and aperiodic activity across scalp and frontoinsular regions with cognitive and symptomatic measurements, we only found a significant relationship between the periodic power at dACC during the eyes‐open condition with composite cognition in general and working memory in particular.

The pronounced increase in theta power in SSD found in this study aligns with the substantial body of research reporting elevated low‐frequency oscillations in this population (Hirano and Uhlhaas 2021; Newson and Thiagarajan 2018). Beyond global increases, disturbances of theta power and theta‐band connectivity are particularly reported over frontal regions in SSD (Boutros et al. 2008; Kim et al. 2020; Shreekantiah Umesh et al. 2016). Consistent with these findings, we found elevated theta power to be extended in three frontoinsular regions (dACC, R‐INS, and L‐INS), with the most robust association observed between increased theta power in the dACC and worse working memory, as measured by the digit sequencing task, across both eyes‐closed and eyes‐open conditions, though only the latter survived correction for multiple comparisons. Moreover, elevated dACC theta power was also linked to global cognitive impairment, marked by a lower BACS composite score. Theta power, specifically within the ACC, is considered a key mechanism supporting cognitive control and efficient neural communication of relevant information, which is essential for both working memory and global cognition (Lett et al. 2014; McLoughlin et al. 2022). Our findings suggest an abnormal increase in dACC theta power during resting‐state EEG may serve as a potential cognitive marker, particularly when spectral power is separated from underlying aperiodic activity.

In addition to theta power alterations, we did not observe any other differences in periodic power across other frequency bands (alpha, beta, or low‐gamma power). Unlike theta power, which frequently shows elevations in SSD, findings regarding gamma activity are more heterogeneous. Some studies suggest that gamma amplitude measured at resting state decreases as the disorder progresses to chronic stages, proposing it as a potential marker of disease progression (Grent‐'t‐Jong et al. 2018), while others report increased gamma power (Baradits et al. 2019), or no significant differences at all (Hirano et al. 2015). Disturbances in the gamma frequency band are largely attributed to the E/I mechanism, specifically due to disruptions in GABAergic activity. GABAergic interneurons are critical for generating and synchronizing gamma oscillations, as they provide inhibitory control over pyramidal neurons and regulate the timing and synchronization needed for gamma activity (Buzsáki and Wang 2012; Shin et al. 2011). Dysfunction in GABAergic pathways can disrupt this balance, leading to disorganized and dysregulated gamma oscillations, potentially indicating changes in the E/I balance (Ahmad et al. 2022).

In contrast to all previous studies which did not consider the removal of aperiodic signal from the spectral power, this study was specifically focused on aperiodic‐adjusted low‐gamma power. However, across all frequency bands, gamma power estimates were less reliable, where less than half of the participants showed activity in this range. This is attributed to the small peaks outside the alpha and beta frequency bands that do not pass the algorithm threshold for being detected as true oscillatory activity (Donoghue et al. 2020). Taken together, these findings suggest that while gamma abnormalities may reflect underlying disruptions in E/I balance, their inconsistent expression (especially after adjusting for aperiodic components) highlights the need for more refined methodological approaches and caution when interpreting gamma power as a robust biomarker in SSD.

Another marker often reported in the SSD population, also replicated by our findings, is the decrease of alpha peak frequency (Murphy and Öngür 2019; Ramsay et al. 2021; Yeum and Kang 2018). This marker is closely related to global cognition, with disturbances in visual processing being a major contributor (Ramsay et al. 2021). In our SSD sample, we observed uncorrected significant associations between slower alpha center frequency and impaired performance in motor tasks, executive functioning, verbal fluency, and overall cognitive performance as captured by global scalp activity. While these findings did not survive correction for multiple comparisons and should be interpreted with caution, they align with previous literature suggesting that alpha slowing might reflect cognitive dysfunction across diagnostic categories. Alpha center frequency is known to be flexible and responsive to stimulation or cognitive load, yet also demonstrates strong test–retest reliability and trait‐like stability at rest (Ahn et al. 2019; Grandy et al. 2013; Smit et al. 2006). Rather than being a disorder‐specific marker, slowing of alpha center frequency is considered to be a transdiagnostic indicator of cognitive impairment, as it is also observed in cognition‐affected conditions such as dementia, Alzheimer's disease, autism spectrum disorder, and ADHD (Dickinson et al. 2017; Puttaert et al. 2021; Vollebregt et al. 2015). Additionally, age is another independent factor influencing shifts in the alpha center frequency, which, as also shown in this study, decreases with advancing age irrespective of the diagnostic group (Park et al. 2024; Scally et al. 2018).

Evidence regarding aperiodic EEG features in schizophrenia remains scarce and inconsistent, in both findings and employed methodology. Among studies conducted in people with SSDs, five studies have reported no difference (Boudewyn et al. 2025; Earl et al. 2024; Jacob et al. 2023; Racz, Farkas, Stylianou, et al. 2021; Racz, Farkas, Becske, et al. 2025), one reported a decrease (Spencer et al. 2023), and two reported an increase in aperiodic exponent in people with SSDs compared to HC samples (Molina et al. 2020; Peterson et al. 2023). Alterations in aperiodic activity, especially in the aperiodic exponent, are thought to reflect the E/I dynamics of the brain, as demonstrated in both in vivo and in silico studies, where a steeper exponent indicates greater inhibition, while a flatter exponent suggests increased overall excitation (Gao et al. 2017; Wiest et al. 2023). Supporting this, inhibitory agents like propofol steepen the exponent, whereas ketamine's excitatory net effects flatten it (Waschke et al. 2021).

Following these studies, the increase in aperiodic exponent observed in our SSD cohort may reflect an overall shift toward inhibitory activity. Moreover, this effect appeared to be modulated by medication dosage, as higher CPZeq levels were associated with steeper exponents. Within the SSD group, we observed uncorrected negative associations (*p* < 0.05, but not surviving FDR correction) of both exponent and offset with positive symptoms. The same uncorrected association remained even when controlling for medication dosage, suggesting that medication may influence but does not fully explain the observed patterns. This may indicate that a steeper exponent reflects a compensatory mechanism in SSD, possibly influenced by, but not solely dependent on, medication effects. Increasing inhibitory activity in major depression patients via electroconvulsive therapy led to a steeper aperiodic exponent and reduced symptom severity (Smith et al. 2023), suggesting that changes in inhibitory tone may track symptom severity in disorders marked by E/I imbalances. Nonetheless, given the lack of significance after multiple comparison correction, these findings should be regarded as preliminary. Further research with larger samples and longitudinal designs is needed to determine whether aperiodic EEG features serve as reliable markers of excitation/inhibition balance and clinical symptoms in SSD, and whether these relationships are specific to certain diagnostic subgroups within the broader SSD spectrum.

### 4.1 Limitations

Although the signal‐to‐noise ratio of our EEG data was satisfactory, we further tried to enhance our source localization accuracy by providing individual anatomical head models, which are known to improve spatial precision (Michel and Brunet 2019), and by utilizing a beamforming approach, which enables more accurate source estimation for deeper regions (Backus et al. 2016; Quraan et al. 2011; Westner et al. 2022). However, the use of a 32‐channel EEG system may still limit the spatial precision of source‐localized activity, particularly in deeper regions such as the anterior insula (Asadzadeh et al. 2020). Therefore, our source‐level results should be interpreted with caution. We encourage future studies to use high‐density M/EEG to more accurately investigate frontoinsular activity in SSD and to test whether similar source‐level profiles, as well as relationships with cognitive and clinical data, can be replicated.

At the scalp level, we averaged EEG features across all electrodes (if more than 50% of electrodes showed detectable activity for a given participant) to derive global indices of periodic and aperiodic activity. While this approach provides a summary measure, it inherently limits spatial resolution and prevents region‐specific interpretations. Although the aperiodic activity was relatively uniformly distributed across the scalp (see Figure S3), electrode‐wise variability may still carry topographical relevance that is obscured by averaging.

Another limitation to be considered is that although our sample size was relatively large, our SSD group represents only a pool of the disease's phenotypic spectrum, reflecting shared mechanisms across psychotic disorders rather than SSD‐specific disease dynamics. Furthermore, while we interpreted some of our results in the framework of E/I imbalances, we acknowledge that the absence of direct neurobiological measures, such as neurotransmitter concentrations or other neurochemical indices, limits the strength of these conclusions. In addition to that, the characterization of frontoinsular regions in this study followed a hypothesis‐driven approach; however, future studies with higher spatial resolution and statistical power should incorporate control anatomical regions in order to capture the complexity of functional alterations in SSD. Future large‐scale multimodal studies integrating M/EEG with other neuroimaging approaches will be crucial to further our understanding of these results and refine their clinical implications.

## 5 Conclusion

In summary, the present study provides evidence for widespread alterations in both periodic and aperiodic resting‐state EEG activity in individuals with SSD. These alterations are characterized by distinct regional and spectral patterns and show differential associations with cognitive impairment and symptom severity. Periodic activity, particularly theta power, was significantly elevated in SSD compared to HC both globally and across the three frontoinsular regions. Notably, only increased theta power in the dACC was significantly associated with worse overall cognition and lower working memory. In contrast, alpha center frequency was reduced in SSD at global and L‐INS levels, but did not show a significant relationship with cognitive or symptomatic measures. For the aperiodic component, both exponent and offset were higher in SSD at the global level, and their increase was associated with higher CPZeq levels. However, no relationships with cognition or symptom severity survived correction for multiple comparisons. These findings underscore the value of separating periodic and aperiodic components in EEG analyses to better understand the neurophysiological mechanisms underlying SSD. While preliminary, the aperiodic exponent may represent a promising marker of altered excitation–inhibition dynamics, potentially modulated by antipsychotic medication, and should be explored further in larger, longitudinal studies.

## Author Contributions

**Genc Hasanaj:** conceptualization, data curation, formal analysis, methodology, visualization, writing – original draft, writing – review and editing. **Iris Jaeger:** conceptualization, data curation, methodology, writing – original draft, writing – review and editing. **Berkhan Karsli:** formal analysis, methodology, visualization, writing – original draft, writing – review and editing. **Enrico Schulz:** methodology, writing – review and editing. **Emanuel Boudriot:** writing – review and editing. **Lukas Roell:** writing – review and editing. **Maxim Korman:** data curation, writing – review and editing. **Marcel S. Kallweit:** data curation, writing – review and editing. **Fanny Dengl:** data curation, writing – review and editing. **Nicole Klimas:** data curation, writing – review and editing. **Kristin Fischer:** writing – review and editing. **Katharina Hanken:** data curation, writing – review and editing. **Verena Meisinger:** data curation, writing – review and editing. **Joanna Moussiopoulou:** data curation, project administration, writing – review and editing. **Vladislav Yakimov:** data curation, project administration, writing – review and editing. **Susanne Karch:** writing – review and editing. **Alkomiet Hasan:** writing – review and editing. **Andrea Schmitt:** project administration, resources, writing – review and editing. **Peter Falkai:** resources, writing – review and editing. **CDP‐Working Group:** writing – review and editing. **Oliver Pogarell:** writing – review and editing. **Florian J. Raabe:** funding acquisition, project administration, resources, writing – review and editing. **Elias Wagner:** funding acquisition, project administration, resources, writing – review and editing. **Matin Mortazavi:** conceptualization, formal analysis, investigation, methodology, writing – original draft, writing – review and editing. **Daniel Keeser:** funding acquisition, investigation, methodology, project administration, resources, writing – original draft, writing – review and editing.

## Conflicts of Interest

E.W. was invited to advisory boards from Recordati, Teva, and Boehringer Ingelheim; A.H. was a member of advisory boards of Boehringer Ingelheim, Lundbeck, Janssen, Otsuka, Rovi, and Recordati and received paid speakership by these companies as well as by AbbVie and Advanz. He is the editor of the German schizophrenia guideline; P.F. received paid speakership by Boehringer‐Ingelheim, Janssen, Otsuka, Lundbeck, Recordati, and Richter and was a member of advisory boards of these companies. All other co‐authors report no conflict of interest.

## Peer Review

The peer review history for this article is available at https://www.webofscience.com/api/gateway/wos/peer‐review/10.1111/ejn.70263.

## Supporting information


**Figure S1:** Topographic distribution of periodic activity percentage across theta, alpha, beta, and low‐gamma bands, grouped by resting‐state condition (eyes open vs. eyes closed) and diagnostic status (healthy controls vs. schizophrenia spectrum disorders). Black dots show the position of the electrodes. Notably, periodic activity is most pronounced in the alpha and beta bands across both conditions and groups.
**Figure S2:** Barplot showing distribution of periodic EEG activity across three source‐localized regions (L‐INS, R‐INS, dACC) across theta, alpha, beta, and low‐gamma frequency bands, grouped by resting‐state condition (eyes open vs. eyes closed) and diagnostic status (healthy controls vs. schizophrenia spectrum disorders). L‐INS: left anterior insula. R‐INS: right anterior insula. dACC: dorsal anterior cingulate cortex.
**Figure S3:** Topographic distribution of the aperiodic activity (exponent and offset parameters) shown across electrodes for each condition (eyes open vs. eyes closed) and diagnostic group (Healthy Controls vs. Schizophrenia Spectrum Disorder) Black dots show the electrode‐placement.

## Data Availability

Data and code used for analysis in this paper will be shared by the lead contact upon request.
