# Supplementary material for: Periodic and Aperiodic Alterations of Resting‐State EEG in Schizophrenia Spectrum Disorders: Cognitive and Clinical Insights
Source: Eur J Neurosci. 2025 Oct 4;62(7):e70263. doi: 10.1111/ejn.70263 (PMC12495554; doi:10.1111/ejn.70263)
Supplement: Supplementary file 1 — Figure S1: Topographic distribution of periodic activity percentage across theta, alpha, beta, and low‐gamma bands, grouped by resting‐state condition (eyes open vs. eyes closed) and diagnostic status (healthy controls vs. schizophrenia spectrum disorders). Black dots show the position of the electrodes. Notably, periodic activity is most pronounced in the alpha and beta bands across both conditions and groups. Figure S2: Barplot showing distribution of periodic EEG activity across three source‐localized regions (L‐INS, R‐INS, dACC) across theta, alpha, beta, and low‐gamma frequency bands, grouped by resting‐state condition (eyes open vs. eyes closed) and diagnostic status (healthy controls vs. schizophrenia spectrum disorders). L‐INS: left anterior insula. R‐INS: right anterior insula. dACC: dorsal anterior cingulate cortex. Figure S3: Topographic distribution of the aperiodic activity (exponent and offset parameters) shown across electrodes for each condition (eyes open vs. eyes closed) and diagnostic group (Healthy Controls vs. Schizophrenia Spectrum Disorder) Black dots show the electrode‐placement. [file EJN-62-0-s001.docx]

## **Supplemental Methods**

### **EEG data preprocessing**

An automated ICA preprocessing pipeline was utilized for preprocessing resting-state EEG. The pipeline is a slightly modified version of the preprocessing pipeline used in the paper by [(Adams et al., 2022)](https://www.zotero.org/google-docs/?lIGPS6), which is implemented in MATLAB (The Mathworks Inc.) using EEGLAB v2022.0 [(Delorme & Makeig, 2004](https://www.zotero.org/google-docs/?vLQp96))(<https://sccn.ucsd.edu/eeglab/>). Considering that both the eyes-open and eyes-closed conditions were recorded in a single session, the data was initially segmented into their respective conditions based on their event markers. First, the data was re-referenced to the mastoid bones (A1 and A2) and then downsampled to 256 Hz. A data filtering was applied, setting the frequency range between 1 to 70 Hz, along with a notch filter from 49.5 to 50.5 Hz. The data was further segmented into 6-second epochs.
 Specific procedures were followed to remove artefacts. First, epochs with a mean greater than ± 5 standard deviations were removed. Additionally, epochs containing linear trends (using pop_rejtrend, max slope=5, min R^2^=0.7), low-frequency bands (<2 Hz) within the power threshold of -50 to 50 dB, and high-frequency bands (20-40 Hz) within the power threshold of -100 to 25 dB (using pop_rejspec) were excluded. Subsequently, the removal of bad channels was performed (using pop_rejchan) based on their extreme values on the power spectrum (-4 to 6 std), kurtosis (-7 to 15 std), and joint probability (-9 to 7 std). In cases where more than 50% of the epochs were rejected at this point, the same rejection process was repeated, but with channel rejection performed first and epoch rejection second. If still more than 50% of the data was rejected, or if more than 20% of electrodes were removed, the dataset was discarded for both conditions. Otherwise, the preprocessing continued with the Independent Component Analysis (ICA) and detected artifact components were removed using the Multiple Artefact Rejection Algorithm (MARA) [(Winkler et al., 2011)](https://www.zotero.org/google-docs/?bBkySs). After MARA, a second round of epoch and channel rejection was applied with slight changes in channel removal parameters for the spectrum (-6 to 5 std), kurtosis (-6 to 9), and joint probability (-7 to 7). Bad or missing channels were then interpolated, and the epoched data were re-converted to continuous data (using eeg_eeg2continuous) for further analyses.
 The sample for this study was derived from a larger pre-processed dataset, consisting of participants with high-quality EEG data recorded from 32 electrodes. In the final sample, the mean electrode rejection rate for healthy controls (HC) was 4.62% during the eyes-closed condition, compared to 5.04% for individuals with schizophrenia spectrum disorder (SSD). In the eyes-open condition, the channel rejection rate was 2.54% for HC and 2.84% for SSD participants. For epoch rejection, HC had 4.74% of epochs removed during the eyes-closed condition, while SSD participants had 6.93% removed. In the eyes-open condition, 5.69% of epochs were removed for healthy controls, compared to 7.41% for SSD participants.

### **EEG source localization**

After the data were preprocessed, we performed source localization using Brainstorm software (Version: April-2024) [(Tadel et al., 2011)](https://www.zotero.org/google-docs/?tAQNEv). Initially, the anatomical data for each participant, preprocessed for every participant (skull stripped, segmented, surface generated) using FreeSurfer v7.3.2 [(Dale et al., 1999; Fischl, 2012)](https://www.zotero.org/google-docs/?5tIsyB) (<http://surfer.nmr.mgh.harvard.edu/>), were imported in the Brainstorm, which were matched with the EEG data for all the participants. For every participant, default electrode positions were added using the ICBM 152 template, to ensure proper electrode positioning based on 10-20 electrode placement [(Fonov et al., 2011)](https://www.zotero.org/google-docs/?fIy06P). All the electrodes were projected into the scalp. Subject-specific Boundary Element Method (BEM) surfaces were generated, using 1922 vertices for each layer (scalp, outer skull, and inner skull), with a thickness of 4mm applied to the skull [(Gramfort et al., 2010; Kybic et al., 2005)](https://www.zotero.org/google-docs/?fBMPNP). After BEM, the head model was computed for each subject using the *process_headmodel* function with OpenMEEG [(Gramfort et al., 2010)](https://www.zotero.org/google-docs/?uHF6Pu) using the cortex surface as the source space. The standard three layers of scalp, skull, and brain were selected, with their respective standard conductive values of 1, 0.0125, and 1. An adaptive integration is used to refine the mesh in regions where greater accuracy was needed. Noise covariance was computed across the entire recording, using an identity matrix (identity = 1) to assume uniform noise across all channels, helping filter out noise during source localization. The EEG data were processed in blocks (dcoffset = 1) to account for slow drifts, enhancing the robustness of the noise model. Similarly, data covariance was calculated over the whole time window (datatimewindow = [0, maxTime]) directly from the EEG recordings (identity = 0), also processed in blocks to capture slow trends accurately. For source estimation, we employed the LCMV beamformer approach using Brainstorm’s process_inverse_2018 function [(Van Veen et al., 1997; Westner et al., 2022)](https://www.zotero.org/google-docs/?RBj76U). Dipole orientations were set to unconstrained. Noise was modeled using the median method with regularization (0.1), and the signal-to-noise ratio (SNR) was computed using the root mean square (rms) method with an SNR threshold of 1e-06. After computing source-localization, the activity is extracted for each of the regions of interests (ROIs/scouts). The preprocessed data were then reconstructed into an EEGLAB *.set* file for each participant, with the activity of each ROI/scout as a single channel.

## **Supplemental Figures**


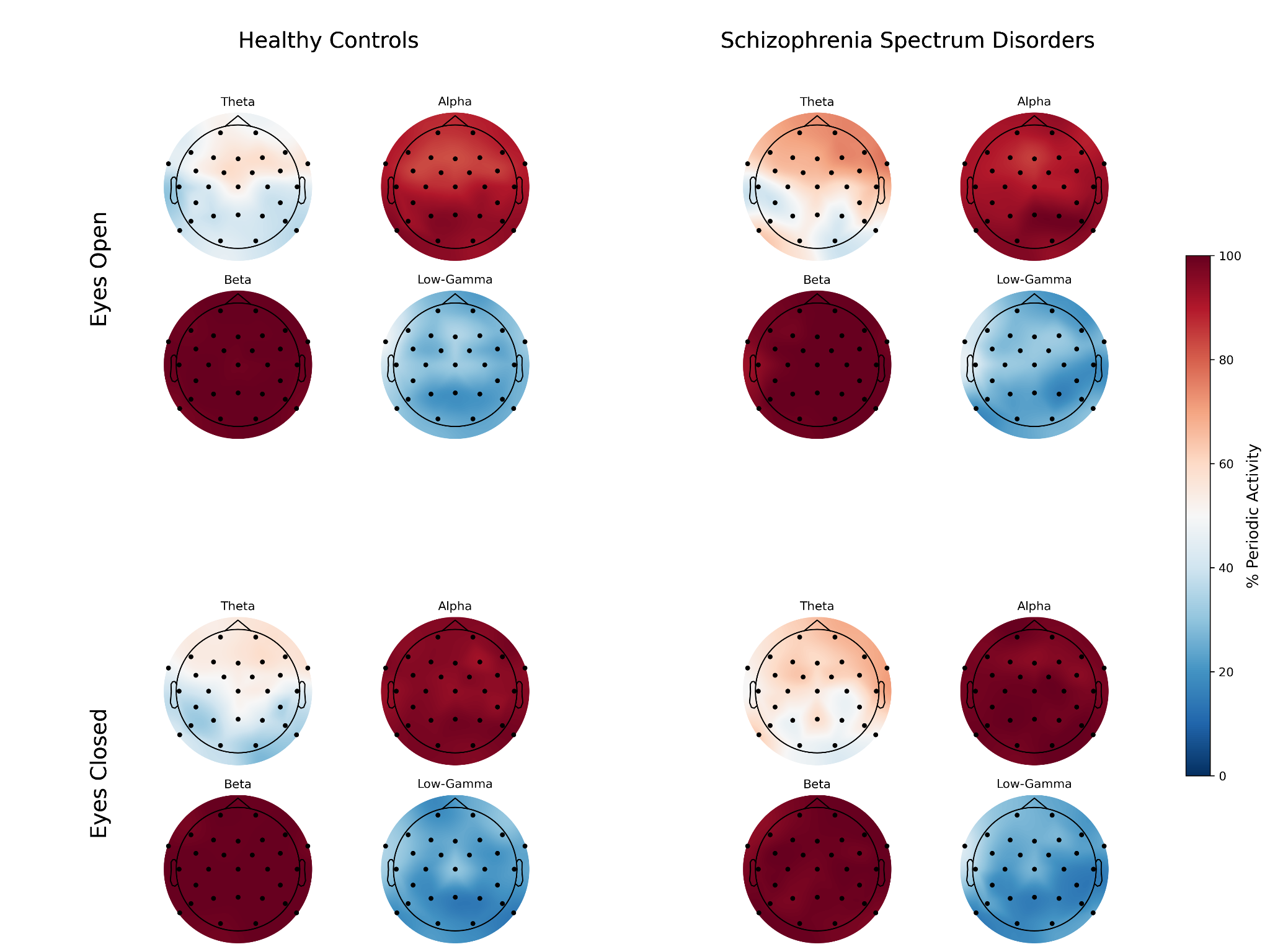


### **Figure S1.** Topographic distribution of periodic activity percentage across theta, alpha, beta, and low-gamma bands, grouped by resting-state condition (eyes open vs. eyes closed) and diagnostic status (healthy controls vs. schizophrenia spectrum disorders). Black dots show the position of the electrodes. Notably, periodic activity is most pronounced in the alpha and beta bands across both conditions and groups.

###
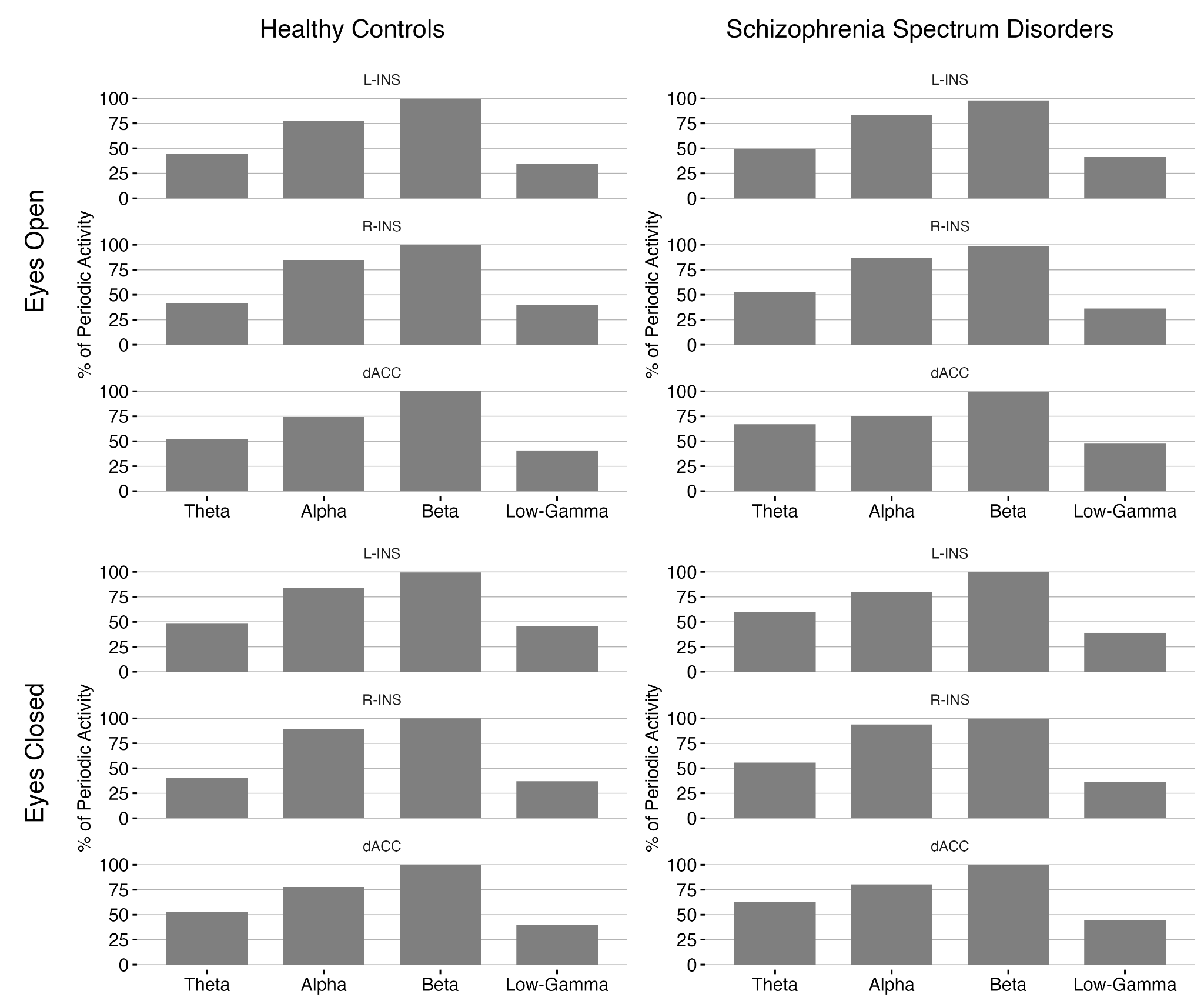


### **Figure S2.** Barplot showing distribution of periodic EEG activity across three source-localized regions (L-INS, R-INS, dACC) across theta, alpha, beta, and low-gamma frequency bands, grouped by resting-state condition (eyes open vs. eyes closed) and diagnostic status (healthy controls vs. schizophrenia spectrum disorders). L-INS: left anterior insula. R-INS: right anterior insula. dACC: dorsal anterior cingulate cortex.
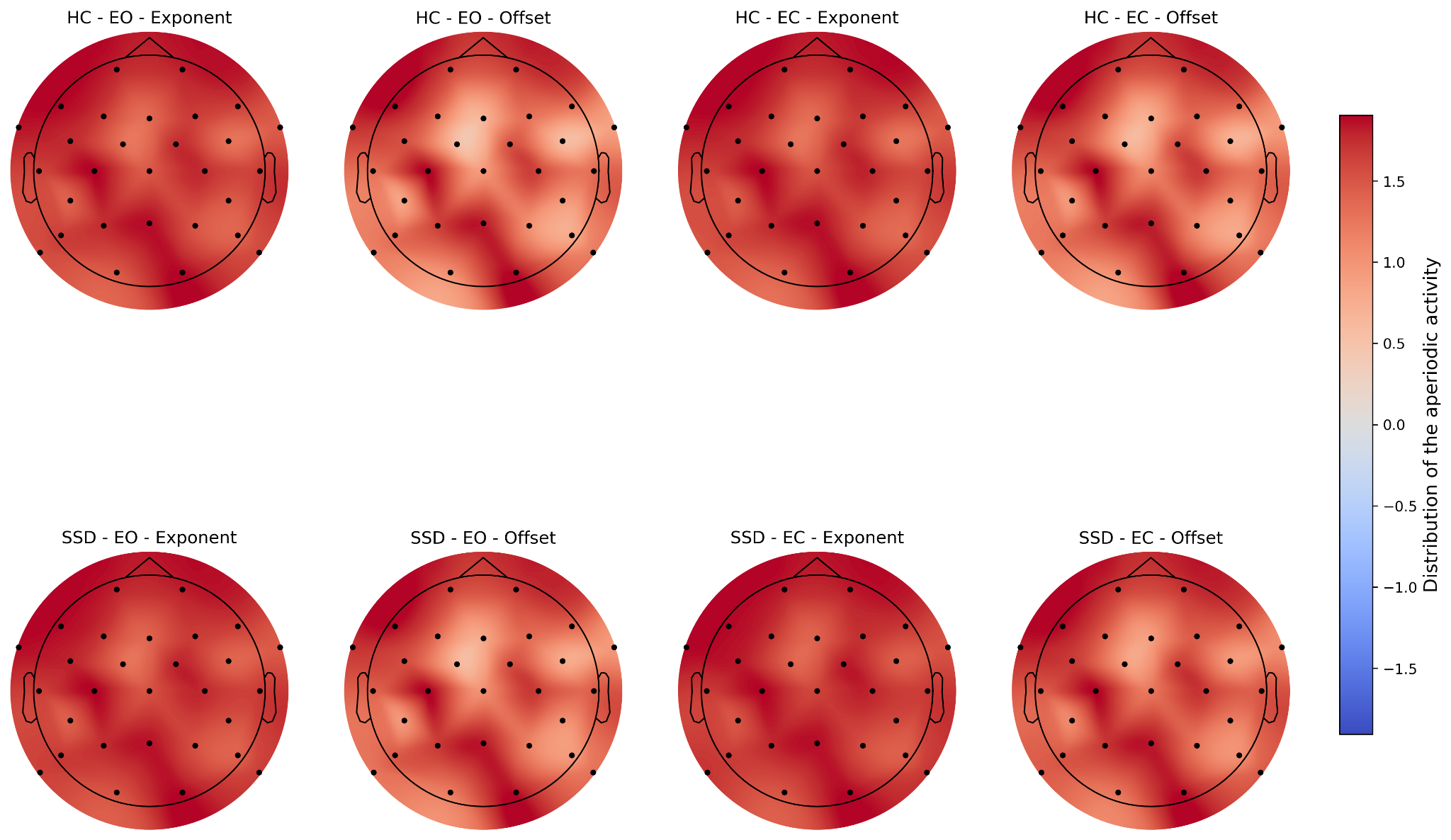


### **Figure S3**. Topographic distribution of the aperiodic activity (exponent and offset parameters) shown across electrodes for each condition (eyes open vs. eyes closed) and diagnostic group (Healthy Controls vs. Schizophrenia Spectrum Disorder) Black dots show the electrode-placement.

###

## **Supplemental References**

[Adams, R. A., Pinotsis, D., Tsirlis, K., Unruh, L., Mahajan, A., Horas, A. M., Convertino, L., Summerfelt, A., Sampath, H., Du, X. M., Kochunov, P., Ji, J. L., Repovs, G., Murray, J. D., Friston, K. J., Hong, L. E., & Anticevic, A. (2022). Computational Modeling of Electroencephalography and Functional Magnetic Resonance Imaging Paradigms Indicates a Consistent Loss of Pyramidal Cell Synaptic Gain in Schizophrenia. *Biological Psychiatry*, *91*(2), 202–215. https://doi.org/10.1016/j.biopsych.2021.07.024](https://www.zotero.org/google-docs/?9V9RlU)

[Dale, A. M., Fischl, B., & Sereno, M. I. (1999). Cortical surface-based analysis. I. Segmentation and surface reconstruction. *NeuroImage*, *9*(2), 179–194. https://doi.org/10.1006/nimg.1998.0395](https://www.zotero.org/google-docs/?9V9RlU)

[Delorme, A., & Makeig, S. (2004). EEGLAB: An open source toolbox for analysis of single-trial EEG dynamics including independent component analysis. *Journal of Neuroscience Methods*, *134*(1), 9–21. https://doi.org/10.1016/j.jneumeth.2003.10.009](https://www.zotero.org/google-docs/?9V9RlU)

[Fischl, B. (2012). FreeSurfer. *NeuroImage*, *62*(2), 774–781. https://doi.org/10.1016/j.neuroimage.2012.01.021](https://www.zotero.org/google-docs/?9V9RlU)

[Fonov, V., Evans, A. C., Botteron, K., Almli, C. R., McKinstry, R. C., & Collins, D. L. (2011). Unbiased average age-appropriate atlases for pediatric studies. *NeuroImage*, *54*(1), 313–327. https://doi.org/10.1016/j.neuroimage.2010.07.033](https://www.zotero.org/google-docs/?9V9RlU)

[Gramfort, A., Papadopoulo, T., Olivi, E., & Clerc, M. (2010). OpenMEEG: Opensource software for quasistatic bioelectromagnetics. *BioMedical Engineering OnLine*, *9*(1), 45. https://doi.org/10.1186/1475-925X-9-45](https://www.zotero.org/google-docs/?9V9RlU)

[Kybic, J., Clerc, M., Faugeras, O., Keriven, R., & Papadopoulo, T. (2005). Fast multipole acceleration of the MEG/EEG boundary element method. *Physics in Medicine & Biology*, *50*(19), 4695. https://doi.org/10.1088/0031-9155/50/19/018](https://www.zotero.org/google-docs/?9V9RlU)

[Tadel, F., Baillet, S., Mosher, J. C., Pantazis, D., & Leahy, R. M. (2011). Brainstorm: A user-friendly application for MEG/EEG analysis. *Computational Intelligence and Neuroscience*, *2011*, 879716. https://doi.org/10.1155/2011/879716](https://www.zotero.org/google-docs/?9V9RlU)

[Van Veen, B. D., van Drongelen, W., Yuchtman, M., & Suzuki, A. (1997). Localization of brain electrical activity via linearly constrained minimum variance spatial filtering. *IEEE Transactions on Bio-Medical Engineering*, *44*(9), 867–880. https://doi.org/10.1109/10.623056](https://www.zotero.org/google-docs/?9V9RlU)

[Westner, B. U., Dalal, S. S., Gramfort, A., Litvak, V., Mosher, J. C., Oostenveld, R., & Schoffelen, J.-M. (2022). A unified view on beamformers for M/EEG source reconstruction. *NeuroImage*, *246*, 118789. https://doi.org/10.1016/j.neuroimage.2021.118789](https://www.zotero.org/google-docs/?9V9RlU)

[Winkler, I., Haufe, S., & Tangermann, M. (2011). Automatic Classification of Artifactual ICA-Components for Artifact Removal in EEG Signals. *Behavioral and Brain Functions*, *7*(1), 30. https://doi.org/10.1186/1744-9081-7-30](https://www.zotero.org/google-docs/?9V9RlU)
